# Seroprevalence of *Theileria equi* and *Babesia caballi* in horses in Spain

**DOI:** 10.1051/parasite/2017015

**Published:** 2017-05-12

**Authors:** Maria Guadalupe Montes Cortés, José Luis Fernández-García, Miguel Ángel Habela Martínez-Estéllez

**Affiliations:** 1 Parasitology and Parasitic Diseases, Animal Health Department, Veterinary Faculty, University of Extremadura 10071 Cáceres Spain; 2 Genetics and Animal Breeding, Veterinary Faculty, University of Extremadura 10071 Cáceres Spain

**Keywords:** Equine piroplasmoses, Spain, Seroprevalence, IFAT, cELISA

## Abstract

Equine piroplasmoses are enzootic parasitic diseases distributed worldwide with high incidence in tropical and subtropical regions. In Spain, there is insufficient epidemiological data about equine piroplasmoses. The main aim of the present study was therefore to estimate the prevalence of *Theileria equi* and *Babesia caballi* in five regions and obtain information about the risk factors. This study was conducted in the central and south-western regions of Spain, using indirect fluorescence antibody testing (IFAT) in 3,100 sera samples from apparently healthy horses of different ages, breeds, coat colours, genders and geographical locations. The overall seroprevalence was 52%, consisting of 44% seropositive for *T. equi* and 21% for *B. caballi*. There was a significant association between age (*p* < 0.0001), breed (*p* < 0.004), geographical location (*p* < 0.0001) and the seroprevalence, but neither the coat colour nor the gender was significantly associated with prevalence. In addition, it was proved that most of the geographic areas showed a moderate to high prevalence. The statistical *κ* value was used to compare the results obtained by the IFAT and the competitive enzyme-linked immunosorbent assay (cELISA) utilised to test some samples (*n* = 108) and showed a higher concordance for *T. equi (κ* = 0.68) than for *B. caballi* (*κ* = 0.22). Consequently, this revealed the importance of developing an appropriate technique to detect each haemoparasite.

## Abstract (fr)

Les piroplasmoses équines sont des parasitoses enzootiques cosmopolites qui existent surtout dans les régions tropicales et subtropicales. En Espagne, peu de données épidémiologiques sont disponibles sur les piroplasmoses équines. Ainsi, le principal objectif de la présente étude a été d’estimer la séroprévalence de l’infection par *Theileria equi* et *Babesia caballi* dans cinq régions et obtenir des informations sur les facteurs de risque. Cette étude a été menée dans les régions du centre et du sud-ouest de l’Espagne en utilisant le test d’immunofluorescence indirecte (IFI) sur un échantillon de 3100 sérums de chevaux apparemment sains de différents âges, races, robes, sexe et origines géographiques. La séroprévalence globale était de 52 %, y compris 44 % séropositifs pour *T. equi* et 21 % pour *B. caballi*. Il y avait une association significative entre l’âge (*p* < 0.0001), la race (*p* < 0.004), la localisation géographique (*p* < 0.0001) et la séroprévalence. Cependant, ni la couleur du pelage ni le sexe n’avaient de corrélation significative avec la séroprévalence. En outre, la plupart des régions étaient dans un état de prévalence modérée à élevée. Le coefficient de concordance *κ* a été utilisé pour comparer les résultats obtenus par IFI et cELISA pour tester 108 échantillons, et a montré une concordance plus élevée pour *T. equi* (*κ* = 0.68) que pour *B. caballi* (*κ* = 0.22). Cela démontre l’importance de développer une technique appropriée pour détecter chaque hémoparasite.

## Introduction

Equine piroplasmoses (EPs) are important and widespread tick-borne diseases in horses. This parasitic disease affects all equid species including horses, donkeys, mules and zebras. Two species of parasites, *Babesia caballi* (Nuttall and Strickland 1910) and *Theileria equi* (formerly *Babesia equi*, Laveran 1901), cause this infection. These protozoa parasitise erythrocytes and they can co-infect animals [18, 65, 83]. The disease is characterised by a variety of symptoms such as fever, anaemia, jaundice, haematuria and lymphadenopathy [32]. The initial acute phase can cause death, but the survivor animals become carriers and reservoirs of infection for vector ticks [28]. Therefore, large economic losses are generated due to the treatments, the decrease in performance of the animals or the negative impact on international trade [31, 56].

In Spain, EPs are enzootic diseases [13, 31] as they have been diagnosed in autochthonous horses for decades [21, 22, 41–43] but there is insufficient epidemiological information about this disease and its vectors in Spain.

Several diagnostic methods are used to detect the infection, such as microscopic examination of stained blood smears, which is useful in the acute phase of infection onset, though serological techniques are better in order to identify chronic carriers. These techniques include the complement fixation test (CFT), the indirect fluorescent antibody test (IFAT), and the competitive enzyme-linked immunosorbent assay (cELISA), which utilises the *EMA-1* protein and a specific monoclonal antibody (MAb) to detect *T. equi,* and the recombinant *RAP-1* protein and an MAb reactive with a peptide epitope of a 60 KDa *B. caballi* antigen to diagnose the other parasite. The last two tests are recommended by the World Organisation for Animal Health (OIE) for the serodiagnosis of EP [73]. The diagnosis of these haemoprotozoan infections can be carried out using molecular assays such as conventional single PCR [13], multiplex PCR [7, 96], nested PCR [72, 78, 95] or real-time PCR [54]. Thus, the combination of two or more of these methods is currently recommended to diagnose the EP [102].

The main goal of this survey was to estimate the seroprevalence and geographic distribution of EP in central and southwest Spain. In fact, it is the largest study that has been conducted in Spain. It is intended to identify areas in which to implement more effective control measures against both the pathogens and their vectors. In addition, we analysed 108 randomly selected sera samples to compare concordance of the two serological methods most often used in the diagnosis of this parasitic infection that affects equids in Spain: the indirect fluorescence antibody test (IFAT) vs. an immunoenzymatic assay (cELISA). This study helped to further understand the situation of the Purebred Spanish Horse with regard to these infections in this emblematic and autochthonous breed.

## Materials and methods

### Sampled animals and area of study

**Figure 1.**
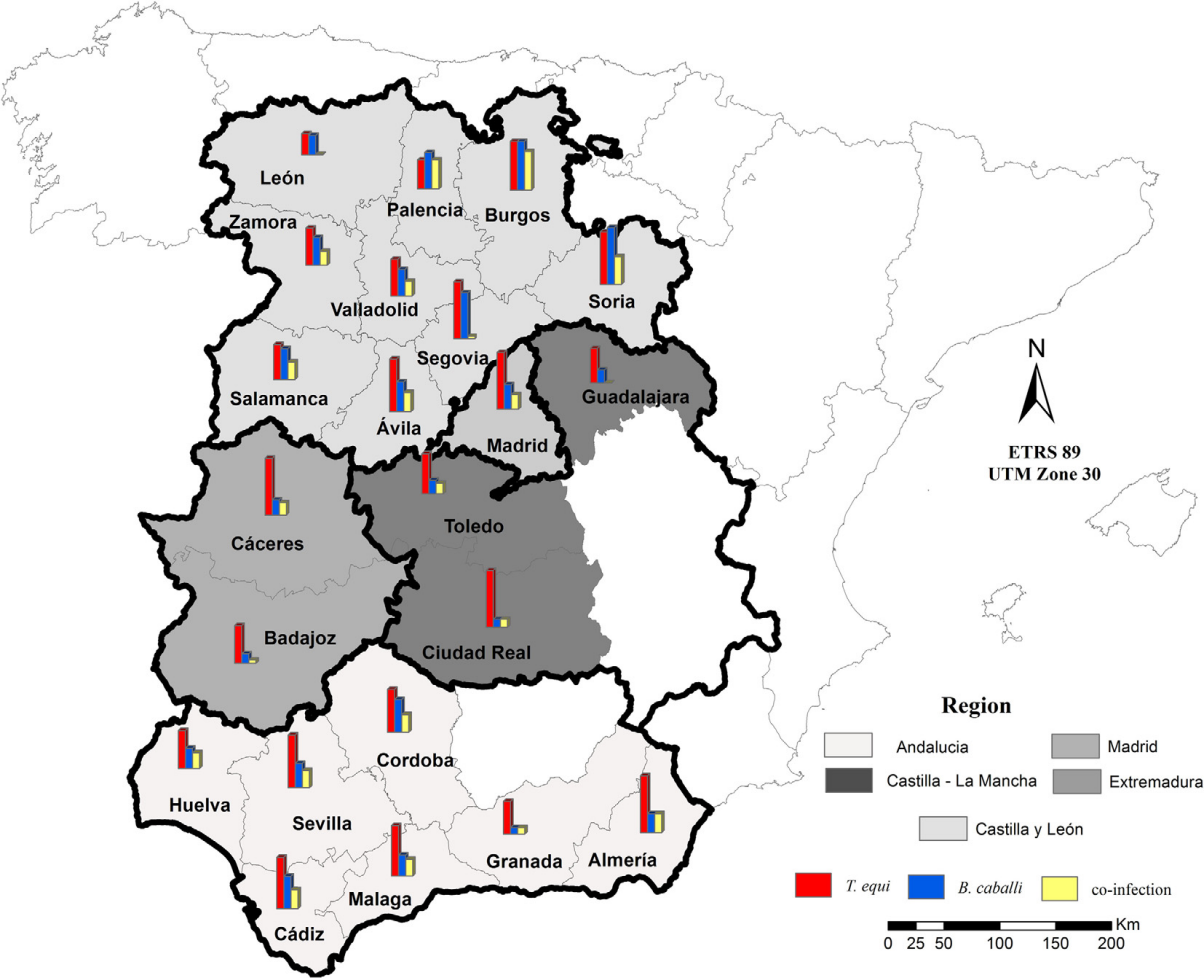
Map of equine piroplasmosis prevalence by region in Spain. The histogram within each province represents the positive horses using percentages.

This study was carried out between February and September 2014 in various regions of Spain: Andalusia, Castilla-La Mancha, Castilla-León, Extremadura and Madrid (Fig. 1). Blood samples were collected from horses’ jugular veins into sterile vacuum tubes with and without anticoagulant. Plasma and serum samples were obtained by centrifugation at 4° C at 2500 rpm for 10 min and were stored at −20° C until testing. The plasma and sera were used for the IFAT and the cELISA, respectively.

This study included 3100 animals (1309 females and 1791 males) with no clinical signs of piroplasmoses between 9 months and 30 years of age (mean age: 7.5 years). Different breeds were tested including the Spanish Pure Breed horse, Anglo-Arabian, Arabian horse, Balearic horse, Hanoverian horse, Lusitano, Thoroughbred, Selle Français and crossbred horses. Information on aptitude was annotated; thus, most of the animals were breeding horses though there were saddle horses (recreation or sports). Data were studied according to recorded information sent by owners and/or veterinarians: gender, breed, age, geographical origin and coat colour.

Win Episcope 2.0 was used [99] to estimate the minimal sample size needed to guarantee the validity of this study. According to the equine census data obtained in 2013 from MAGRAMA (“Ministerio de Agricultura, Alimentación y Medio Ambiente”, Spain) [61] in each region studied (Table S1), at least 381 animals from each area were sufficient in order to detect a 50% prevalence of subclinical EP infection with a certainty of 95% [89]. However, in the region of Madrid, the sample size was smaller than necessary (*n* = 312) (Fig. 1, Table S1).

### Indirect fluorescence antibody test (IFAT) and immunoenzymatic assay (cELISA)

The IFAT was used for the detection of antibodies against *T. equi* and *B. caballi*. The antigen was obtained from naturally infected horses with a parasitaemia higher than 3%. Both protocols to prepare the *T. equi* and the *B. caballi* antigen and the assay were conducted as described by Camacho et al. [21]. The slides were examined under the fluorescence microscope (Leica DMLS^®^) at a magnification of 400 (10 × 40). Positive and negative sera were included in each run as controls.

The cELISA test was carried out with commercially available test kits (VMRD, Inc. Pullman, WA, USA) to detect antibodies against *T. equi* and *B. caballi*. These tests were conducted following the manufacturer’s instructions. The plates were read on a plate reader (Multiskan Ascent, Thermo Electron Corporation^®^) at an optical density of 620 nm. Samples associated with percent inhibition (PI) values <40% were considered negative, while if the PI value was ≥40%, sera were considered positive.

The IFAT and the cELISA techniques are the most useful methods to diagnose equine piroplasmoses. For this reason, a comparison between the techniques was needed since it had not been done previously in Spain. Thus, from the 3100 serum samples tested by IFAT, 108 samples were randomly selected and tested by cELISA.

### Statistical analysis

The seroprevalences of *T. equi*, *B. caballi* and co-infection relative to certain characteristics (age, breed, coat colour, gender and geographical location) were determined at the 95% confidence interval (CI). These epidemiological data were compared with the IFAT results using a logistic regression-binary (LR-binary). Animals were considered as units of analysis for determining the significance of association. Data analyses were performed using Statistical Package for Social Sciences (SPSS) 11.0 software for Windows. The odds ratios were calculated at a 95% confidence interval (95% CI). Tests with a *p*-value ≤ 0.05 were considered statistically significant.

Due to the semi-quantitative characteristic of age as a variable, it was evaluated by both (i) exploring the difference in means between categories in the IFAT variable, and (ii) using it as an ordinal variable, then using the OR calculated as the average value of each risk factor compared to the previous in descending order. The statistical significance of seroprevalence between pairs of regions (Castilla-La Mancha, Andalusia, Castilla-León, Extremadura and Madrid) was calculated using a non-parametric test with the Monte Carlo Method. In order to determine the concordance between the two serological techniques (the IFAT and the cELISA), Cohen’s *κ* test was used [24]. The *κ* values < 0 indicate no agreement and values between 0 and 0.20 indicate slight agreement, 0.21–0.4 fair agreement, 0.41–0.60 moderate, 0.61–0.80 substantial and 0.81–1 almost perfect agreement.

## Results

### Seroepidemiological study

The serological examination of 3100 horses by IFAT showed that the overall seroprevalence of the equine piroplasmoses in southwest Spain was 52.45% (*SE =* 0.009). Of the 3100 tested samples, 1381 sera (44.55%; *SE =* 0.009) were positive for *T. equi*, 643 samples (20.74%; *SE =* 0.007) were positive for *B. caballi* and 398 horses (12.84%; *SE =* 0.006) had antibodies against both parasites.

The seroprevalence in horses from Castilla-La Mancha was the highest (67.54%) (significance calculated by the Monte Carlo method [95% CI]; *p* < 0.001). The prevalence results in horses from Andalusia, Castilla-León and Extremadura (50.94%; 53.62% and 51.5%, respectively) were not statistically significant (significance calculated by the Monte Carlo method [95% CI] *ns*). The lowest seroprevalence was observed in Madrid (38.14%) (significance calculated by the Monte Carlo method [CI 95%]; *p* < 0.001) (Table 1). The mean age by region was as follows: 6.24 (*SE =* 0.183), 7.15 (*SE =* 0.250), 10.25 (*SE =* 0.320), 6.43 (*SE =* 0.161) and 8.26 (*SE =* 0.384) years for Andalusia, Castilla-La Mancha, Castilla-León, Extremadura and Madrid, respectively.

**Table 1.** Prevalence of *T. equi* and *B. caballi* antibodies (by IFAT) in horses from different regions of Spain

Region	Seroprevalence	*T. equi* (% ± *SE*)	*B. caballi* (% ± *SE*)	Co-infection (% ± *SE*)	Total (% ± *SE*)
Andalusia	Males	241/560 (43.04 ± 0.021)	105/560 (18.75 ± 0.017)	77/560 (13.75 ± 0.014)	269 (48.04 ± 0.021)
	Females	175/394 (44.42 ± 0.025)	107/394 (27.16 ± 0.022)	65/394 (16.50 ± 0.019)	217 (55.07 ± 0.025)
	Overall	416 (43.61 ± 0.016)	212 (22.22 ± 0.013)	142 (14.88 ± 0.011)	486 (50.94 ± 0.016)
Castilla-La Mancha	Males	125/207 (60.39 ± 0.034)	42/207 (20.29 ± 0.028)	34/207 (16.43 ± 0.026)	133 (64.25 ± 0.033)
	Females	114/175 (65.14 ± 0.036)	37/175 (21.14 ± 0.031)	26/175 (14.86 ± 0.027)	125 (71.43 ± 0.034)
	Overall	239 (62.57 ± 0.025)	79 (20.68 ± 0.021)	60 (15.71 ± 0.019)	258 (67.54 ± 0.024)
Castilla-León	Males	165/424 (38.92 ± 0.024)	130/424 (30.66 ± 0.022)	62/424 (14.62 ± 0.017)	233 (54.95 ± 0.024)
	Females	103/266 (38.72 ± 0.030)	85/266 (31.95 ± 0.029)	51/266 (19.17 ± 0.024)	137 (51.50 ± 0.031)
	Overall	268 (38.84 ± 0.019)	215 (31.16 ± 0.018)	113 (16.38 ± 0.014)	370 (53.62 ± 0.019)
Extremadura	Males	167/391 (42.71 ± 0.025)	61/391 (15.60 ± 0.018)	34/391 (8.70 ± 0.014)	194 (49.61 ± 0.025)
	Females	190/371 (51.21 ± 0.026)	32/371 (8.63 ± 0.015)	23/371 (6.20 ± 0.013)	199 (53.64 ± 0.026)
	Overall	357 (46.85 ± 0.018)	93 (12.20 ± 0.012)	57 (7.48 ± 0.010)	393 (51.67 ± 0.018)
Madrid	Males	68/209 (32.54 ± 0.032)	24/209 (11.48 ± 0.022)	15/209 (7.18 ± 0.018)	77 (36.84 ± 0.033)
	Females	33/103 (32.04 ± 0.046)	20/103 (19.42 ± 0.039)	11/103 (10.68 ± 0.031)	42 (40.78 ± 0.049)
	Overall	101 (32.37 ± 0.027)	44 (14.10 ± 0.020)	26 (8.33 ± 0.016)	119 (38.14 ± 0.028)

(*SE* = Standard Error).

In this study, it has been shown that *T. equi*, *B. caballi* and mixed infection were detected across regions. Notably, the parasite detected most frequently was *T. equi*, ranging from 62.57 to 32.37% in Andalusia and Madrid, respectively (Table 1). However, *B. caballi* prevalence was half as high, ranging from 31.16 to 12.20% in Castilla-León and Extremadura, respectively (Table 1). The maximum of mixed infections was 16.38% in Castilla-León (Table 1). The regional seroprevalence for male and female horses is shown in Table 1 (see also Table S2 for province details).

Approximately half of the tested Spanish Pure Breed horses were seropositive (50.89%) consisting of 41.69% horses *T. equi* seropositive and 16.32% seropositive for *B. caballi*. Meanwhile, 9.90% of the tested horses were positive for both parasites.

Regarding the goodness of the LR-binary model (χ^2^ = 372.93; *p* < 0.0001 and *B* = 0.098 (*SE* = 0.038); *p* < 0.006), age (*p* < 0.0001), breed (*p* < 0.004) and geographical location (*p* < 0.0001) were significant, explaining between 0.155 (Cox and Snell’s *R*^2^) and 0.207 (Nagelkerke’s *R*^2^) of the dependent variable (seroprevalence), with 62.4% of the cases correctly classified. This indicates that the model is acceptable. Other risk factors, the coat colour and the gender, were not significant and fall out of the model (Tables S3 and S4 for *T. equi* and *B. caballi*, respectively, for details). The difference between average age of the positive and the negative horses (6.92 [95% CI: 6.61–7.22] subtracted from 8.19 [95% CI: 7.86–8.53]) by the *T. equi* IFAT test was 1.27 years. On the basis of these results, non-overlap between the CIs of the average ages supported statistically significant differences in the age of seropositivity, based on the IFAT test. Moreover, the OR values between a specific age and the previous one were slightly similar but significantly increased from three to 11 years of age. This was accompanied by a change of significance regarding the percentages in seroprevalence, reaching a threshold of around 44–65% of positivity from eleven years of age. Regarding the breed, OR values are significantly different among non-native breeds such as Arabian horses, Thoroughbred, Selle Français and crossbreeds, which always showed OR values higher than those from the autochthonous breeds like Spanish Pure Breed horses for *T. equi*. Focusing on the geographical distribution, it was observed that there was a higher risk of *T. equi* infection in Extremadura and Castilla-León since the OR was significant.

A similar result was obtained for *B. caballi*, since neither coat colour nor gender was risk factor for this parasitic infection. The difference between the average age for seronegative and seropositive horses was 1.75 years (7.14 [95% CI: 6.90–7.39], that is the mean age of seronegative horses, subtracted from 8.89 [95% CI: 8.31–9.57], that is the mean age of seropositive through the IFAT test). This difference was slightly higher than that for *T. equi*. The infection risk increased until 12 years of age; from there, the seropositivity settled around 20–45% and there were no significant differences of seroprevalence above 12 years of age. However, the OR between a specific age group and the previous one for *B. caballi* was about three times higher than for *T. equi,* but turnover (more frequent negativity change to more frequent positivity) in favour of positivity from the nine-year threshold occurs only for *T. equi* (Table S3). There was a significant association between the breeds Arabian horses and Thoroughbred, and seropositivity for *B. caballi*. The seropositivity rate in Extremadura was significantly lower than in other regions.

### IFAT vs. cELISA results

The concordance between the techniques was similar. Of the 108 tested sera samples, both diagnostic methods showed concordant results for *T. equi* in 91 sera (84.26%), meanwhile for *B. caballi* the same results were observed in 89 samples (82.41%). Focusing on the anti-*T. equi* antibodies, 7 sera were positive by IFAT but were found negative by the cELISA, and 10 serum samples were negative by IFAT but positive by cELISA. Analysing the *B. caballi* results, it was found that 16 horses had antibodies for that parasite by IFAT but did not show reactivity in the cELISA, and three animals were negative by IFAT but positive by cELISA (Table 2).

**Table 2.** Serological results by IFAT and cELISA for *T. equi* (A) and *B. caballi* (B), respectively.

		cELISA		
		Positive	Negative	Total
*T. equi* (A)				
IFAT	Positive	48	7	55
	Negative	10	43	53
	Total	58	50	108
*B. caballi* (B)				
IFAT	Positive	4	16	20
	Negative	3	85	88
	Total	7	101	108

The concordance between the two serological methods for *T. equi* using the *κ* coefficient was 0.68. According to Landis and Koch’s rating scale for the *κ* index, there was substantial agreement between the techniques. A fair agreement (Cohen’s *κ* = 0.22) was observed between the techniques for *B. caballi*.

## Discussion

Equine piroplasmoses are diseases that affect a large number of horses worldwide. Spain is an enzootic zone; therefore, information about the prevalence of this infection in horse populations is essential to control the disease and to reduce the economic losses generated. Different serological tests are available for epidemiological studies (IFAT, cELISA) [56, 58]. Currently, both techniques are recommended by the World Organisation for Animal Health for importation [73]. This study mainly used the IFAT and 108 randomly selected samples were analysed using both methods. Importantly, survey samples were collected from a large area in Spain, which made it possible to estimate the overall prevalence in a more realistic manner. Several studies on the seroprevalence of EPs in Spain have been published, although most of them were not as extensive as the present survey. These diseases are widespread in Spain and seroprevalence is high, as it has been reported by other authors [21, 22, 35, 41, 43].

Recently, a survey carried out in all of Spain showed a *T. equi* seroprevalence of 21.9%, a prevalence of 5% for *B. caballi* and co-infection in 2.71% of the tested animals using the cELISA [22]. In the Andalusia region, García-Bocanegra et al. [35] reported a slightly higher seroprevalence for *T. equi* (48.6% vs. 43.61% in the present survey), but the *B. caballi* prevalence was 7.9% in the former, while in the present study it was 22.22%. In 2005, Camacho et al. [21] in Galicia (northwest Spain) estimated the seropositivity for *B. caballi* to be 28.3% in healthy horses using IFAT, which was similar to our results. The difference could be explained by variations in abiotic factors and tick fauna distribution. Furthermore, the particular results for *B. caballi* may also be explained by the use of different diagnostic techniques. Thus, in the present study, it was revealed that the agreement between the cELISA and the IFAT was poorer for *B. caballi*. This difference could be due to the fact that IFAT slides were made with an autochthonous strain, while the commercial cELISA kit used a *RAP-1* foreign antigen, leading to differences in the specificity and the sensitivity of the techniques. As we did, Camacho et al. [21] used the same IFAT technique, which led to a more accurate comparison among regions using the data from both studies. The prevalence estimated in other countries with IFAT or cELISA was different from the present survey. The EP prevalence was higher than in Spain in countries such as Colombia (≥90%) [98], Brazil 78.8% and 65.7% for *T. equi* and *B. caballi*, respectively, [44] or 97.5% for EP [100] and Mongolia with 82.3% EP seroprevalence [16], or 78.8% for *T. equi* and 65.7% for *B. caballi*, respectively [84]. However, it was lower in countries such as the UAE (33.3%) [48], Sudan (25.2%) [85], Portugal (17.9% and 11.1% for *T. equi* and *B. caballi*, respectively) [80], Turkey (18.4–18.5%) [51, 90], Jordan (14.6%) [2], Greece (11.6%) [56], Saudi Arabia (10.4% and 7.5% for *T. equi* and *B. caballi*, respectively) [6], Italy (8.5%) [40], Switzerland (7.3%) [92], the Netherlands (4%) [19] and Korea (1.1%) [87]. In other studies, the *T. equi* seroprevalence was higher than that described in Spain, but the seropositivity for *B. caballi* was lower, this is the case for France (from 58% to 80% for *T. equi* and from 1.2% to 12.9% for *B. caballi*) [33, 38] and Iran (48% and 2%) [1]. Meanwhile, a lower prevalence of *T. equi* was described in Hungary (32%) [30], northern Italy (12.4%) [67] and the Azores Islands (9.1%) [11]. The EP seroprevalence discrepancies could be related to housing conditions, grazing and activity of horses [38, 56, 100]. Also, the measures for control of these diseases, the selected test for the diagnosis [2, 35, 56, 67], the climate and the tick fauna could be important. Thus, temperature and/or humidity and/or precipitation could increase or decrease tick populations [56, 94–96].

Using the IFAT, the *T. equi* seroprevalence was higher than that of *B. caballi*. Different trends were observed by other authors using different techniques (Table 3). *T. equi* was the predominant parasite in 82.14% of the studies in respect to *B. caballi*, but after excluding two studies [60, 81] due to discrepancies between diagnostic methods regarding the predominant haemoparasite.

**Table 3.** *T. equi* and *B. caballi* prevalence by different diagnostic methods, including geographical distribution and predominant parasite.

Diagnostic method	Continent	Country	Sample size	Prevalence in % (*T*. equi vs. *B*. caballi)	Predominant parasite	References
CFT	America	Brazil	582	28.5 and 54.6	*B. caballi* > *T. equi*	Kerber et al. 2009 [53]
	Europe	France	443	58 and 12.9	*T. equi* > *B. caballi*	Guidi et al. 2015 [38]
ELISA	Africa	Egypt	88	14.8 and 0	*T. equi* > *B. caballi*	Mahmoud et al. 2016 [62]
		Sudan	158	63.5 and 4.4	*T. equi* > *B. caballi*	Salim et al. 2008 [85]
	America	Brazil	47	81 and 90	*B. caballi* > *T. equi*	Xuan et al. 2001 [104]
		Brazil	582	26.6 and 69.6	*B. caballi* > *T. equi*	Kerber et al. 2009 [53]
		Brazil	198	78.3 and 69.2	*T. equi* > *B. caballi*	Vieira et al. 2013 [100]
		Costa Rica	130	88.5 and 69.2	*T. equi* > *B. caballi*	Posada-Guzmán et al. 2015 [75]
		Venezuela	360	50.3 and 70.5	*B. caballi* > *T. equi*	Mujica et al. 2011 [69]
		Venezuela	694	14 and 23.2	*B. caballi* > *T. equi*	Rosales et al. 2013 [81]
	Asia	China	70	40 and 24.3	*T. equi* > *B. caballi*	Xuan et al. 2002 [105]
		China	111	34 and 32	*T. equi* > *B. caballi*	Xu et al. 2003 [103]
		China	1990	11.51 and 51.16	*B. caballi* > *T. equi*	Wang et al. 2014 [101]
		UAE	105	32.4 and 4.8	*T. equi* > *B. caballi*	Jaffer et al. 2010 [48]
		India	180	75 and 1.11	*T. equi* > *B. caballi*	Sumbria et al. 2016 [96]
		Japan	2019	2.2 and 5.4	*B. caballi* > *T. equi*	Ikadai et al. 2002 [47]
		Jordan	253	14.6 and 0	*T. equi* > *B. caballi*	Abutarbush et al. 2012 [2]
		Korea	184	1.1 and 0	*T. equi* > *B. caballi*	Seo et al. 2011 [87]
		Mongolia	254	72.8 and 40.1	*T. equi* > *B. caballi*	Boldbaatar et al. 2005 [16]
		Mongolia	250	51.6 and 19.6	*B. caballi* > *T. equi*	Munkhjargal et al. 2013 [70]
		Pakistan	430	41.2 and 21.6	*T. equi* > *B. caballi*	Hussain et al. 2014 [46]
		Thailand	240	5.42 and 2.5	*T. equi* > *B. caballi*	Kamyingkird et al. 2014 [50]
		Turkey	481	16.21 and 0.83	*T. equi* > *B. caballi*	Sevinc et al. 2008 [90]
		Turkey	220	56.8 and 0	*T. equi* > *B. caballi*	Kurt and Yaman 2012 [57]
	Europe	Greece	524	9.2 and 1.1	*T. equi* > *B. caballi*	Kouam et al. 2010 [56]
		Italy	673	39.8 and 8.9	*T. equi* > *B. caballi*	Bartolomé del Pino et al. 2016 [12]
		Portugal	162	17.9 and 11.1	*T. equi* > *B. caballi*	Ribeiro et al. 2013 [80]
		Spain	380	48.6 and 7.9	*T. equi* > *B. caballi*	García-Bocanegra et al. 2013 [35]
		Spain	1067	21.9 and 5	*T. equi* > *B. caballi*	Camino et al. 2016 [22]
IFAT	Africa	Egypt	88	23.9 and 17.0	*T. equi* > *B. caballi*	Mahmoud et al. 2016 [62]
		South Africa	92	97.83 and 52.17	*T. equi* > *B. caballi*	Motloang et al. 2008 [68]
	America	Brazil	93	33.3 and 68.8	*B. caballi* > *T. equi*	Asgarali et al. 2007 [8]
		Brazil	487	91.0 and 83	*T. equi* > *B. caballi*	Heim et al. 2007 [44]
		Mexico	248	45.2 and 27.4	*T. equi* > *B. caballi*	Cantú-Martínez et al. 2012 [23]
	Asia	Iran	100	48 and 2	*T. equi* > *B. caballi*	Abedi et al. 2014 [1]
		Saudi Arabia	241	10.4 and 7.5	*T. equi* > *B. caballi*	Alanazi et al. 2012 [6]
		Thailand	240	8.75 and 5	*T. equi* > *B. caballi*	Kamyingkird et al. 2014 [50]
		Turkey	110	64.5 and 4.5	*T. equi* > *B. caballi*	Akkan et al. 2003 [5]
		Turkey	84	23.8 and 38	*B. caballi* > *T. equi*	Acici et al. 2008 [3]
		Turkey	125	12.8 and 9.6	*T. equi* > *B. caballi*	Karatepe et al. 2009 [51]
		UAE	105	33.3 and 10.5	*T. equi* > *B. caballi*	Jaffer et al. 2010 [48]
	Europe	Italy	412	12.4 and 17.9	*B. caballi* > *T. equi*	Moretti et al. 2010 [67]
		Italy	294	8.2 and 0.3	*T. equi* > *B. caballi*	Grandi et al. 2011 [40]
		Italy	300	41 and 26	*T. equi* > *B. caballi*	Laus et al. 2013 [60]
		Italy	1441	32.2 and 1.9	*T. equi* > *B. caballi*	Sgorbini et al. 2015 [89]
		Netherlands	300	1 and 3	*B. caballi* > *T. equi*	Butler et al. 2012 [19]
		Spain	60	40 and 28.3	*T. equi* > *B. caballi*	Camacho et al. 2005 [21]
		Spain	–	52.5 and 21.3	*T. equi* > *B. caballi*	Habela et al. 2005 [43]
		Switzerland	689	4.4 and 1.5	*T. equi* > *B. caballi*	Sigg et al. 2010 [92]
PCR	Africa	Egypt	88	36.4 and 19.3	*T. equi* > *B. caballi*	Mahmoud et al. 2016 [62]
		South Africa	92	5.43 and 0	*T. equi* > *B. caballi*	Motloang et al. 2008 [68]
		Sudan	131	25.2 and 0	*T. equi* > *B. caballi*	Salim et al. 2008 [85]
		Sudan	499	35.95 and 0	*T. equi* > *B. caballi*	Salim et al. 2013 [86]
		Tunisia	104	11.54 and 0.96	*T. equi* > *B. caballi*	Ros-García et al. 2013 [82]
	America	Brazil	487	59.7 and 12.5	*T. equi* > *B. caballi*	Heim et al. 2007 [44]
		Costa Rica	130	46.2 and 20	*T. equi* > *B. caballi*	Posada-Guzmán et al. 2015 [75]
		Guatemala	74	52 and 48	*T. equi* > *B. caballi*	Teglas et al. 2005 [97]
		Venezuela	136	61.8 and 4.4	*T. equi* > *B. caballi*	Rosales et al. 2013 [81]
	Asia	India	180	14.14 and 0	*T. equi* > *B. caballi*	Sumbria et al. 2016 [96]
		Iran	100	45 and 0	*T. equi* > *B. caballi*	Abedi et al. 2014 [1]
		Iran	240	10.83 and 5.83	*T. equi* > *B. caballi*	Malekifard et al. 2014 [63]
		Jordan	288	18.8 and 7.3	*T. equi* > *B. caballi*	Qablan et al. 2013 [77]
		Korea	224	0.9 and 0	*T. equi* > *B. caballi*	Seo et al. 2013 [88]
		Turkey	200	7 and 3	*T. equi* > *B. caballi*	Güçlü and Karaer 2007 [37]
		Turkey	203	2.96 and 1.97	*T. equi* > *B. caballi*	Kizilarslan et al. 2015 [55]
		Turkey	125	8.8 and 0	*T. equi* > *B. caballi*	Guven et al. 2017 [39]
		Mongolia	192	92.7 and 1.2	*T. equi* > *B. caballi*	Sloboda et al. 2011 [93]
		Mongolia	250	6.4 and 42.4	*B. caballi* > *T. equi*	Mans et al. 2015 [64]
	Europe	Central Balkans	142	22.5 and 2.1	*T. equi* > *B. caballi*	Davitkov et al. 2016 [26]
		France	111	80 and 1.2	*T. equi* > *B. caballi*	Fritz 2010 [33]
		Italy	294	2.72 and 0	*T. equi* > *B. caballi*	Grandi et al. 2011 [40]
		Italy	300	6.0 and 11.7	*B. caballi* > *T. equi*	Laus et al. 2013 [60]
		Italy	263	70.3 and 10.3	*T. equi* > *B. caballi*	Bartolomé del Pino et al. 2016 [12]
		Romania	178	20.3 and 2.2	*T. equi* > *B. caballi*	Gallusová et al. 2014 [34]

Infected horses may remain lifelong carriers of *T. equi,* whereas *B. caballi* is eliminated from the bloodstream 1–4 years post-infection, which could explain the seroprevalence difference for these parasites [12, 28, 85]. This fact could explain why in horses older than nine years, the percentage of infected animals exceeds that of uninfected animals in the case of *T. equi,* which never occurs for *B. caballi*. Furthermore, treatments do not completely eliminate *T. equi* from the animals [18, 28]. The situation reported by other authors is different since *B. caballi* is more prevalent than *T. equi*, which has been related to the presence of the appropriate tick vectors for the transmission of *B. caballi* [69].

Several authors [4, 12, 35, 38, 45, 49, 50, 56, 70, 74, 77, 79, 84, 90, 100] suggested that age was a risk factor, since older animals could have been exposed to ticks for a longer period than young animals. Nevertheless, other authors showed the absence of an age-prevalence relationship [1, 3, 8, 10, 17, 23, 26, 36, 40, 46, 69, 75, 76, 80, 92, 94]. The present study pointed out that less than 1/4 of the foals and yearlings were seropositive for both parasites, with an increase in the percentage of infected horses until stabilisation at 11 and 14 years of age for *T. equi* and *B. caballi,* respectively, as Cantú-Martínez et al. reported [23]. Other studies have also reported that *T. equi* antibodies were higher in older than in young animals [8, 9, 27, 50, 51, 56]. In addition, Vieira et al. [100] indicate that the seroprevalence of *T. equi* increased with age but in contrast, the presence of antibodies to *B. caballi* decreased in the oldest animals, which resembles the pattern described in this study. There is evidence that animals infected with *T. equi* may become lifelong carriers [18]. However, infection with *B. caballi* may also persist in the subclinical state for 1–4 years only. This fact may partially explain our results, whereby *T. equi* seroprevalence remained over 60% from eleven years of age, but *B. caballi* seroprevalence did not exceed the level of 44% in 16-year-old horses in Spain, where these parasites cohabit.

It was found that Spanish breeds have a lower infection prevalence than non-native breeds. Sevinc et al. [90] and Aharonson-Raz et al. [4] recognised that the seroprevalence in Arabian horses was higher, as also found in the present study, especially for *B. caballi*. Bartolomé del Pino et al. [12] indicated that the prevalence in crossbred horses was significantly higher than other (pure) breeds. Other surveys showed no association between infection prevalence and breed [2, 10, 36, 75].

Shkap et al. [91] considered that the differences in prevalence between male and female horses may be due to different management practices for the two sexes. In the present study, however, differences between male and female horses were not observed.

In contrast to Aharonson-Raz et al. [4], no significant association between coat colour and the results of the diagnostic test was observed. Further studies are needed to understand the origin of this difference.

Significantly higher seroprevalence was obtained only in Extremadura and Castilla-León horses. There have also been studies that demonstrated statistically significant differences between counties or regions [2, 3, 8, 12, 16, 26, 29, 35, 51, 52, 56, 87, 91, 94, 96].

With respect to Cohen’s *κ* analysis, the concordance between the IFAT and the cELISA for *T. equi* was higher than for *B. caballi*, showing a fair agreement for *B. caballi*. The *EMA-1* gene of the strains used to make the *T. equi* recombinant antigen in the cELISA and the strains from Spain were probably similar. Consequently, for *B. caballi*, the different results between this technique and cELISA may be related to this fact. However, the *RAP-1* gene of strains used to make the recombinant antigen in the cELISA could be different from the *RAP-1* gene of Spanish strains. Recently, Montes et al. [66] showed one Spanish *B. caballi* strain to be genetically different from that described by Cacciò et al. [20] based on the *β-tubulin* gene. Also, the existence of genetic differences between strains within a country or among countries has been reported previously [14, 25, 71]. These authors showed that there was heterogeneity in the *18S rRNA* gene both for *T. equi* and *B. caballi* in Spain and South Africa. In support of our study and focusing on the *RAP-1* gene of *B. caballi*, Bhoora et al. [15], Rapoport et al. [79] and Mahmoud et al. [62] indicated failure to detect the *B. caballi* parasite. In accordance with Rapoport et al. [79], there could be doubts as to the ability of the cELISA to serve as a sole regulatory test for the international horse trade. The IFAT used in the present survey was performed with Spanish *B. caballi* strains, since it appears that they detect the presence of haemoparasite antibodies more successfully than the cELISA. Thus, Kuttler et al. [59] and Prochno et al. [76] confirmed that, due to regional differences, the use of antigens from autochthonous strains provides the best results.

## Conclusions

The risk factors that seem to be associated with the presence of equine piroplasmoses in Spain are age, breed and geographical location. Meanwhile, coat colour and gender were not significantly associated in these diseases. The seroprevalence in young animals is relatively low, but as horses get older they become seropositive, especially concerning *T. equi*. In addition, the comparison between IFAT and cELISA revealed a possible underestimation of the presence of *B. caballi* when using cELISA.

## Conflict of interest

The authors declare there is no conflict of interest
